# Circulating T Cell Subpopulations Correlate With Immune Responses at the Tumor Site and Clinical Response to PD1 Inhibition in Non-Small Cell Lung Cancer

**DOI:** 10.3389/fimmu.2018.01613

**Published:** 2018-08-03

**Authors:** Nataly Manjarrez-Orduño, Laurence C. Menard, Selena Kansal, Paul Fischer, Bijal Kakrecha, Can Jiang, Mark Cunningham, Danielle Greenawalt, Vishal Patel, Minghui Yang, Ryan Golhar, Julie A. Carman, Sergey Lezhnin, Hongyue Dai, Paul S. Kayne, Suzanne J. Suchard, Steven H. Bernstein, Steven G. Nadler

**Affiliations:** ^1^Bristol-Myers Squibb, Princeton, NJ, United States; ^2^M2GEN, Tampa, FL, United States

**Keywords:** T cells subpopulations, melanoma, lung cancer, checkpoint blockade, PD1 and PDL1

## Abstract

Agents targeting the PD1–PDL1 axis have transformed cancer therapy. Factors that influence clinical response to PD1–PDL1 inhibitors include tumor mutational burden, immune infiltration of the tumor, and local PDL1 expression. To identify peripheral correlates of the anti-tumor immune response in the absence of checkpoint blockade, we performed a retrospective study of circulating T cell subpopulations and matched tumor gene expression in melanoma and non-small cell lung cancer (NSCLC) patients. Notably, both melanoma and NSCLC patients whose tumors exhibited increased inflammatory gene transcripts presented high CD4^+^ and CD8^+^ central memory T cell (CM) to effector T cell (Eff) ratios in blood. Consequently, we evaluated CM/Eff T cell ratios in a second cohort of NSCLC. The data showed that high CM/Eff T cell ratios correlated with increased tumor PDL1 expression. Furthermore, of the 22 patients within this NSCLC cohort who received nivolumab, those with high CM/Eff T cell ratios, had longer progression-free survival (PFS) (median survival: 91 vs. 215 days). These findings show that by providing a window into the state of the immune system, peripheral T cell subpopulations inform about the state of the anti-tumor immune response and identify potential blood biomarkers of clinical response to checkpoint inhibitors in melanoma and NSCLC.

## Introduction

Since their initial approval for the treatment of melanoma in 2014, anti-PD1 agents have transformed cancer therapy, more than doubling median overall survival rates for melanoma (1) and non-small cell lung cancer (NSCLC) (2). It is clear that not every patient or cancer type benefits from an anti-PD1 agent. As the PD1/PDL1 regulatory pathway inhibits the effector activity of T cells, the efficacy of an anti-PD1 agent depends not only on the presence of a counter-ligand to inhibit but also more importantly, on the availability of tumor-specific T cells whose activity can be unleashed by the therapeutic agent (3).

The quest to identify cancer patients who will benefit from therapy includes several companion and complementary diagnostic assays performed on tumor biopsies. These assays aim to identify PDL1 expression in the tumor and tumor microenvironment (4), and tumor mutation burden (TMB) as a surrogate measure of neoantigen availability (5). In recent findings, the presence of an active immune infiltrate, evaluated through the expression of transcripts associated with CD8^+^ T cell function, correlates highly with a positive clinical outcome toward anti-PD1 agents (6).

The determination of a patient’s probability of response to anti-PD1/PDL1 agents is critical to inform a course of treatment and requires the identification of readily assessable biomarkers. While tissue biopsies provide a window into the immune response unfolding within the tumor microenvironment, tumor heterogeneity and the presence of multiple tumor sites can lead to mischaracterization of the magnitude of the anti-tumor immune response (7). In addition, the extent of this response depends on the state of the host’s immune system. Factors such as genetic background, age, gender, and therapies such as chemotherapy and radiotherapy affect the immune system (8). This heterogeneity creates a need to improve the evaluation of the status of the immune system in cancer patients and its associated clinical outcomes. The dynamic nature of tumor evolution in response to therapy means that long-lasting clinical responses require an immune system fit to adapt to this changing environment (9).

An effective immune response toward a tumor requires neoantigen availability (5) and presentation to T cells, and subsequently the entry of antigen-exposed, activated T cells to the tumor. The tumor, in turn, can downregulate the immune response by expressing PDL1, which activates a regulatory mechanism in the T cell through its interaction with PD1 (3).

To determine if blood T cell subpopulations reflect the immune response against the tumor, we performed a cross-sectional, retrospective analysis of peripheral T cells and matched tumor gene expression in melanoma and NSCLC samples collected before checkpoint inhibitors became part of the standard of care. We observed a correlation between the degree of expression of inflammatory transcripts in the tumor and the percentages of circulating central memory (CM) and effector (Eff) CD4^+^ and CD8^+^ T cells, expressed as independent CD4^+^ and CD8^+^ CM/Eff T cell ratios. High CM/Eff T cell ratios correlate with inflamed tumors. Given that tumor inflammation correlates with good clinical response to checkpoint inhibitors, we tested whether high CM/Eff T cell ratios correlate with clinical outcome in a cohort of nivolumab-treated NSCLC patients. In this cohort, those patients with high CM/Eff T cell ratios experienced more prolonged progression-free survival (PFS). Given that melanoma and NSCLC patients with inflamed tumors, as well as NSCLC patients with longer PFS have high CM/Eff T cell ratios, we propose that measurement of these ratios in an easily accessible peripheral blood sample is a convenient biomarker of the state of the T cell arm of the immune system. These findings represent progress in the characterization of peripheral immunity, immune state, and its relationship to the inflammatory status of the tumor.

## Materials and Methods

### Tissues and PBMC

Banked PBMC and matched flash frozen tumor samples from melanoma and NSCLC patients were obtained in collaboration with M2GEN and Moffitt Cancer Center (Tampa, FL, USA) and consented through their Total Cancer Care protocol. Control PBMC were obtained from the Bristol-Myers Squibb employee volunteer blood donation program (Table 1).

**Table 1:** Patient characteristics and demographics.

Cohort	Patients (*n*)	Mean age (SD), years	Male, *n*	Stage II, *n* (%)	Stage III, *n* (%)	Stage IV, *n* (%)
Control	27	54.9 (8.5)	12			
Melanoma	43	63.7 (15.0)	31	1 (2.3)	17 (39.5)	25 (58.1)
Nonsquamous NSCLC	40	65.7 (11.8)	15	16 (4.00)	12 (30.0)	12 (30.0)

For the second NSCLC cohort, we obtained blood samples from 57 patients with NSCLC from a commercial vendor (MT group, CA, USA). A subset of these samples (*n* = 22) are from patients before receiving nivolumab as part of their clinical care. A second blood sample and clinical evaluation was obtained between 8 and 12 weeks after the start of the treatment. Control blood samples were obtained from the BMS employee volunteer blood program and processed simultaneously.

### Flow Cytometry

PBMC were stained for viability with Near Infrared dye (Molecular Probes), blocked and incubated in an antibody mix containing anti-CD127-AF488 (Clone A0195D5), anti-PD1-PE (Clone EH12), anti-CD8-APC-R700 (RPA-T8), anti-CD28-BV650 (CD28.2), anti-CCR7-BV421 (GO43H7), anti-CD25-PECy7 (M-A251), anti-PD-1-PE (EH12), anti-CD45RA-BUV395 (HI100) anti-CD4-BUV495 (SK3), and anti-CD3 BUV737 (SK7).

Whole blood samples were collected and shipped overnight. Whole blood was then stained for viability with Near Infrared dye (Molecular Probes) followed by wash and surface staining with an antibody mix containing: anti-CD45-BV480 (Clone HI30), anti-CD4-AF700 (SK3), anti-CD8-BUV395 (RPA-T8), anti-CD3-BUV496 (UCHT1), anti-CCR7-BV711 (GO43H7), anti-PD-1-APC (MIH4), and anti-CD45RO-BV421 (UCHL1). All samples were read on a BD Fortessa instrument and analyzed with FlowJo. Spanning Tree Progression of Density Normalized Events (SPADE) analysis (10) were implemented on Cytobank (www.cytobank.org). Independent clustering of either CD4^+^ or CD8^+^ T cells used CD45RA, CCR7, and CD28. Both the circles and color scale denote the number of cells in the cluster.

### Gene Expression and Inflammatory Signature

Total RNA was isolated from frozen tumor using AllPrep DNA/RNA/miRNA kit (Qiagen, Valencia, CA, USA) following manufacturer’s recommended protocols. After assessing RNA quality, sequencing libraries were made using the TruSeq Stranded mRNA HT kit (Illumina, San Diego, CA, USA). Libraries were run on an illumina HiSeq 2500 at EA Genomics Services. Paired end FASTQ files were stored in AWS S3, and all analysis took place on AWS EC2 c3.8× large instances created by StarCluster (11).

Gene and isoform expression were calculated using RSEM (12) v1.1.13 and the UCSC hg19 genome annotation. An additional step of calculating gene and isoform quantile normalized read counts was performed using a custom Perl script. Inflammation gene expression scores were calculated based on the gene signature described in Spranger et al. (6) by calculating the mean of the log2, centered normalized data. Genes included in the signature include *CD8A, CCL2, CCL3, CCL4, CXCL9, CXCL10, ICOS, GZMK, IRF1, HLA-DMA, HLA-DMB, HLA-DOA*, and *HLA-DOB*. The scores where then split based on quantiles of the normal distribution as inflamed, intermediate, and non-inflamed.

### Statistics and Visualizations

**Figure 1:**
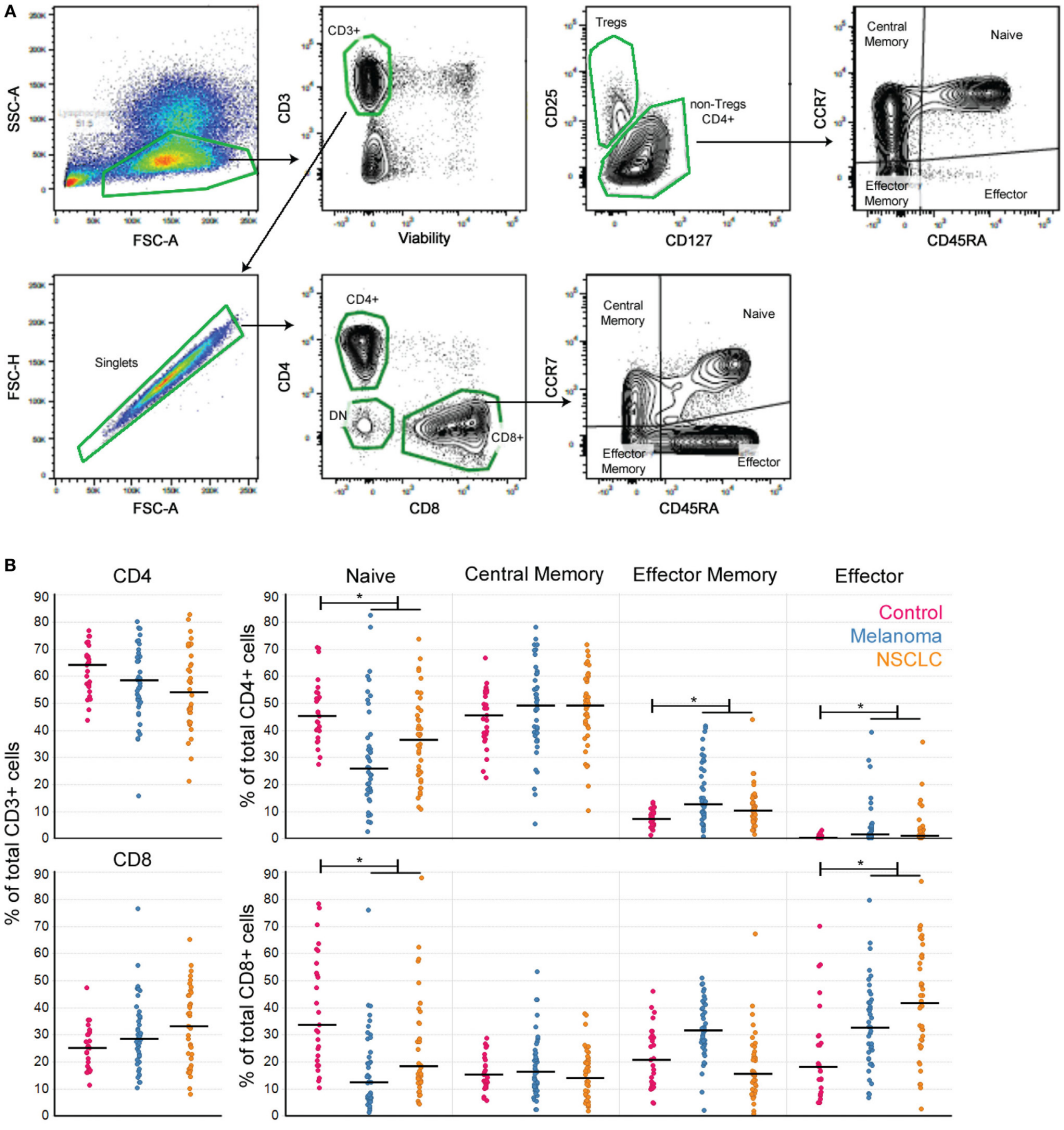

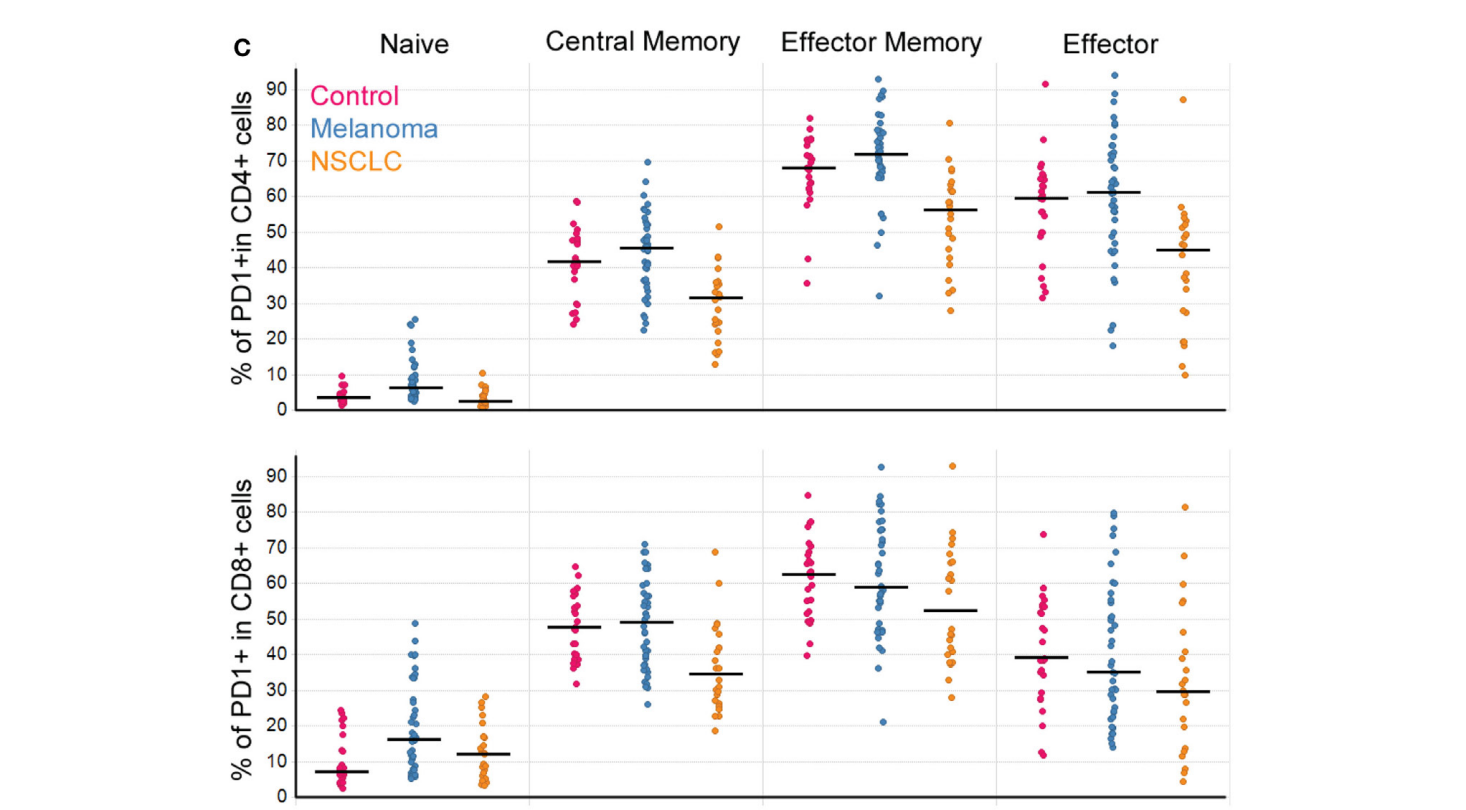
Peripheral T cell subpopulations show evidence of an ongoing immune response in cancer patients. **(A)** Gating strategy to define T cell subpopulations in PBMC. **(B)** CD4^+^ and CD8^+^ T cell subpopulations in PBMC from melanoma and NSCLC patients. **(C)** Percentage of PD1+ cells per T cell subpopulation (controls *n* = 27, melanoma *n* = 43, NSCLC *n* = 40; Bonferroni-corrected *p*-values: *<0.001). Line marks the median.

Comparisons of T cell subpopulations were performed using Student’s *t*-test. For non-normal distributions, data were log-transformed before *t*-test. All reported *p*-values were corrected for multiple comparisons (Figures 1A and 3A).

Fischer’s exact test was used for the analysis of 2 × 2 contingency tables for CM/Eff T cell ratios by inflammation state and PDL1 tumor proportion score (TPS) (separately).

For PFS analysis of patients undergoing treatment with nivolumab, all patients had at least 90 days of follow-up after first dose. PFS was calculated from the first day of nivolumab infusion until physician-confirmed disease progression (clinical or CT confirmed) by a scientist blind to the patient’s biomarkers characteristics. Right-censored data were used to obtain Kaplan–Meier survival estimates and Wilcoxon *p*-values.

All statistical analysis were performed in JMP 13 (SAS, NC).

## Results

### Circulating T Cells in Melanoma and Nonsquamous NSCLC Patients Show Evidence of Ongoing Immune Responses

Patients with cancer have circulating T cells specific for tumor antigens (13). Consequently, we hypothesized that the circulating T cell pool would reflect the immune responses to melanoma and NSCLC. To evaluate this premise in the absence of checkpoint inhibitors, we performed a cross-sectional, retrospective study of T cell subpopulations in archived PBMC from 43 melanoma and 40 NSCLC patients (all of them nonsquamous NSCLC). All of the patients had available matched tumor tissue, and none of them had prior treatment with checkpoint agents (Table 1; Figure 1).

Analysis of T cell subpopulations revealed that as a group, PBMC from cancer patients presented a decrease in the percentages of both CD4^+^ and CD8^+^ naïve T cells, accompanied by an increase in the percentages of EM and Eff CD4^+^ and Eff CD8^+^ T cells compared to control samples (Figure 1B). These findings are consistent with the presence of an ongoing immune response in these patients similar to that observed in patients with autoimmunity (14).

### Association of Circulating T Cell Profiles With the Local Immune Response in Melanoma and Nonsquamous NSCLC

**Figure 2:**
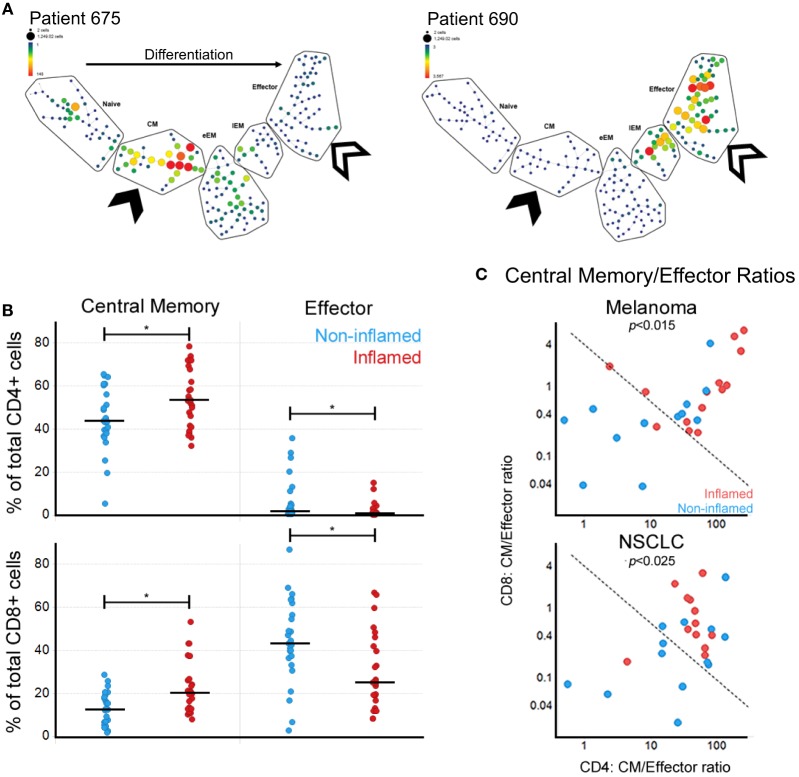
Local immune responses in melanoma and NSCLC correlate with circulating central memory (CM)/effector (Eff) T cell ratios. **(A)** SPADE-generated maturation profiles of CD8^+^ T cells for two melanoma samples showing the divergent patterns of T cell subpopulations. Both have a reduction in naïve CD8^+^ T cells but show an expansion in either CM (solid arrow) or the Eff (open arrow) compartments. eEM, early Effector Memory; EM, Effector Memory. **(B)** Correlation between circulating CM and Eff CD4^+^ and CD8^+^ T cells and tumor inflammation state (*p < 0.05). **(C)** Correlation between CM/Eff T cell ratios by inflammation state in melanoma and NSCLC. Fisher’s exact test *p*-value for a 0.05 significance level. Dividing line generated based on the 90th percentile of controls.

To assess the T cell differentiation patterns present in these patients, we implemented SPADE on the flow cytometry data (see Materials and Methods). Clustering of either CD4^+^ or CD8^+^ T cells using the differentiation markers CD45RA, CCR7, and CD28 showed that in cancer patients, the circulating antigen-experienced T cells present either CM-early Effector Memory or Eff phenotypes (Figure 2A), also reflected by the inverse relationships between CM and Eff subpopulations.

Next, we evaluated how the circulating T cell subpopulations reflect the local immune state observed in the tumors. We used matched frozen tumor tissues to evaluate gene expression profiles of immune-associated genes. We defined the tumors as inflamed, intermediate, and non-inflamed based on quantiles of inflammation gene signature scores. Through further analysis of the inflamed vs. non-inflamed tumors we observed a correlation between tumor inflammation and the percentages of circulating central memory and effector T cells, which while similar in magnitude, showed a different direction. Surprisingly, the peripheral blood populations which showed a positive correlation with inflamed tumors were not the effector T cell subpopulations, but CM, for both CD4^+^ and CD8^+^ T cells (Figure 2B).

Given the inverse relationship between CM and Eff T cells, we calculated CM/Eff ratios for both CD4^+^ and CD8^+^ T cells (Figure 2C). Patients with inflamed tumors by gene expression had a tendency toward high CM/Eff ratios (upper right corner). Interestingly, CM/Eff ratios in patients with high inflammation scores are similar to those of the healthy control samples used in this study. Consequently, we used the 90th percentile of control samples to distinguish between low and high CM/Eff ratios (dotted line), observing that the inflamed melanoma and NSCLC tumors have high CM/Eff ratios compared to those with non-inflamed tumors.

### Circulating CM/Eff T Cell Ratios in NSCLC Are Associated With Longer PFS in Response to Checkpoint Inhibitors

**Figure 3:**
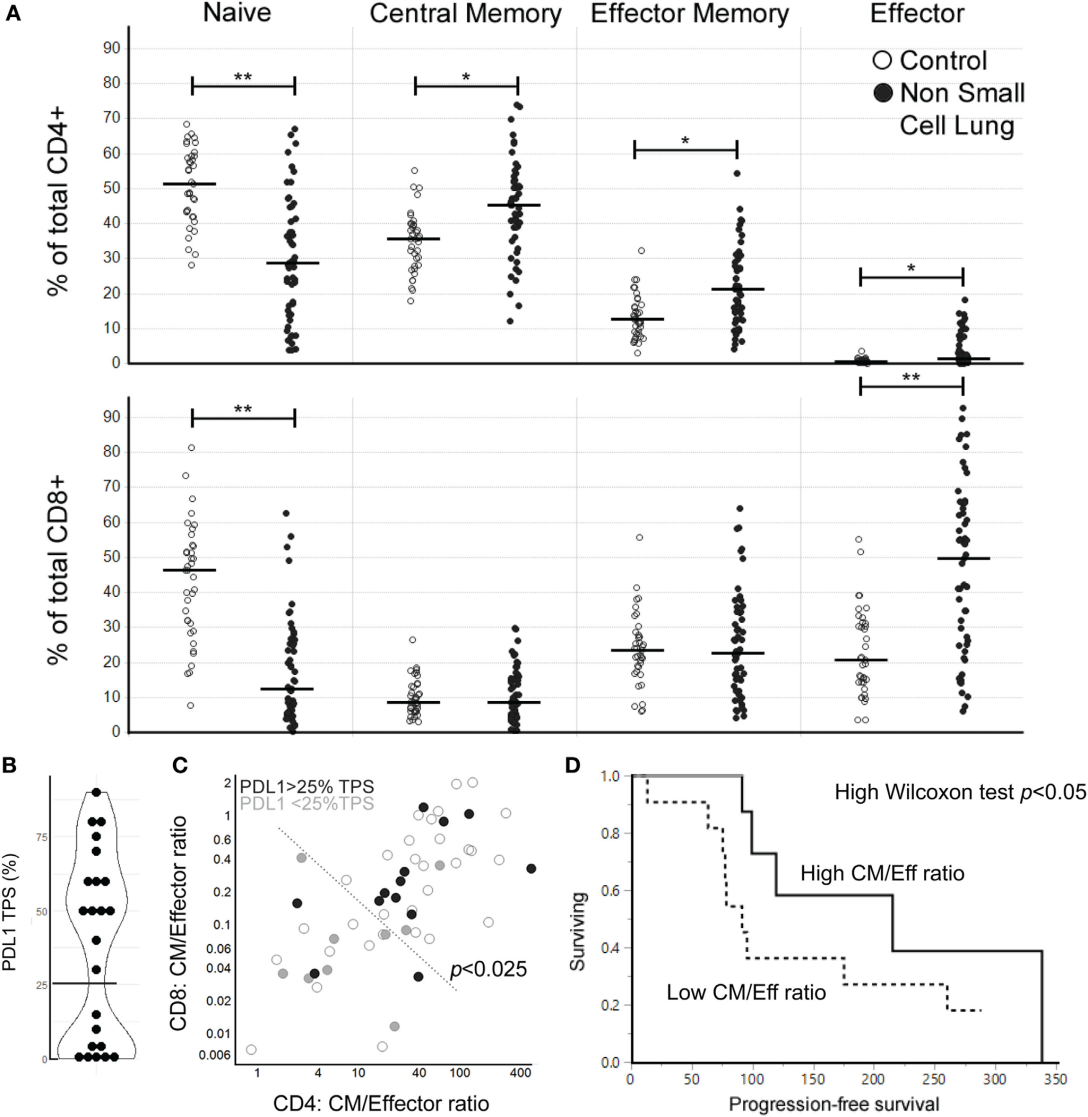

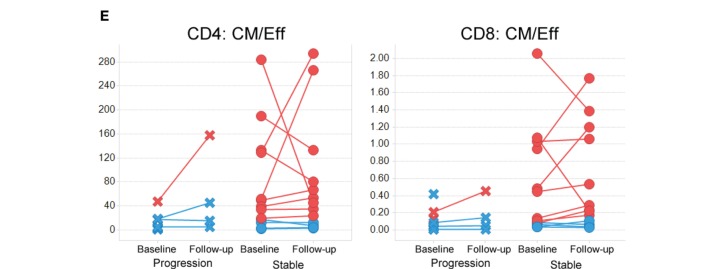
High central memory (CM)/effector (Eff) T cell ratios at baseline are associated with longer progression-free survival (PFS) in response to nivolumab treatment for NSCLC. **(A)** Peripheral T cell profiles in a second cohort of NSCLC and control samples (Bonferroni-corrected *p*-values: *<0.001, **<0.0001, line marks the median for subpopulation). **(B)** Distribution of PDL1 tumor proportion score (TPS) (*n* = 23, the horizontal line marks the cutoff at the antimode: 25% TPS). **(C)** CM/Eff T cell ratios in the NSCLC cohort coded by PDL1 TPS (open circles: PDL1 expression not evaluated). CM/Eff T cell ratios high vs. low division line is drawn using the 90th percentile of the control samples. Fischer’s *p*-value <0.025. **(D)** PFS after nivolumab treatment (*n* = 22) (*p*-value < 0.05, median survival by CM/Eff ratio: low, 91 days; high 215 days). **(E)** Change in CM/Eff T cell ratios three months after nivolumab-treatment initiation. Patients are classified by physician-reported response to treatment at three months.

To evaluate CM/Eff T cell ratios as a tool to evaluate the status of the T cell arm of the immune system, we collected blood from a second cohort of NSCLC patients (*n* = 57). We were able to observe that the reduction of the naïve compartment and the expansion of Eff T cell subpopulations, both in CD4^+^ and CD8^+^ T cells is a reproducible finding (Figure 3A).

We then hypothesized that the interferon gamma produced during anti-tumor immune responses would lead to the upregulation of PDL1, as this is an interferon gamma-induced gene (15). In the 23 patients where PDL1 expression was measured, we observed a bimodal distribution in the percentage of PDL1 tumor proportion score (%TPS) (Figure 3B). This pattern made us divide the patients at the antimode (25% TPS) as PDL1^neg/low^ and PDL1^high^. High CM/Eff T cell ratios, which we had previously found associated with higher inflammatory signature (Figure 2C), correlate with high PDL1 expression in the tumor (Figure 3C, Fisher’s exact *p* < 0.025).

A subset of these NSCLC patients went on to receive nivolumab as part of their clinical care (*n* = 22). Those patients with high CM/Eff T cell ratios at baseline had an extended PFS compared to those patients with low CM/Eff T cell ratios (Figure 3D, Wilcoxon test *p* < 0.05, median survival time “*low”* ratio: 91 days, “*high*” ratio 215 days). A second blood sample, obtained around 3 months after the initiation of nivolumab treatment did not show major changes in CM/Eff T cell ratios in patients categorized as “*low*,” in contrast to those patients classified as “*high*” (Figure 3E). It is important to mention that because of disease progression, only 7 of the 11 “*low*” patients were still in nivolumab treatment, in contrast to 10 of the 11 “high” patients.

## Discussion

Here, we report that high circulating CM/Eff T cell ratios associate with tumor inflammation in melanoma and NSCLC, as well as with increased PDL1 expression at the tumor and longer PFS in response to nivolumab treatment in NSCLC. To the best of our knowledge, this is the first time that circulating T cell subpopulations are proposed as predictive biomarkers of response to checkpoint inhibitors in NSCLC.

The association between higher frequency of CM T cells (CD4 and CD8) and an increased tumor inflammatory profile is congruent with reports that CM T cells are the primary repository of the immunogenic experiences of a lifetime (16, 17). The inverse relationship between the frequency of Eff T cells in circulation and the inflammation signature in the tumor was nevertheless surprising and could reflect the presence of terminally differentiated T cells that are unable to reach the tumor. Rather than reflecting the immune response against the tumor, we hypothesize that CM/Eff ratios are a way to evaluate the status of the immune system. In this model, immune state evaluated by CM/Eff ratios would be associated with the capacity of a subject to mount an immune response against the tumor that checkpoint inhibitors can potentiate. This model is consistent with the high sensitivity of this analysis to detect cancer patients who have inflamed tumors (>90%, Figure 2C). Nevertheless, its low specificity highlights the multifactorial nature of the anti-tumor response, as other factors, such as TMB, also play a role in the anti-tumor response (18).

These findings provide a window into how the status of the immune system affects the anti-tumor response. Extended clinical responses to checkpoint inhibitors depend on the presence of tumor-specific T cells, and the ability of the immune system to co-evolve with the tumor. Thus, the predominant T cell response shifts as the dominant antigen disappears or mutates (9, 19). Under this model, increased immunological pressure toward the tumor (increased inflammation signature) may drive the upregulation of PDL1 as an immunosuppressive tumor-survival mechanism (20), as observed in the patients with high CM/Eff T cell ratios.

These results align with previous reports that the percentages of CD4 and CD8^+^ T cell memory correlate with clinical response in melanoma patients treated with ipilimumab (21, 22). Moreover, a recent analysis of four melanoma patients (two with stable disease, one progressive disease, and one partial response) show an increase of central memory CD4^+^ T cells in the two patients with longer survival times (23). These data are in line with a recent report of peripheral immune cells and its correlation with response to checkpoint inhibitors in melanoma which also found an association between increased CD8^+^ CM T cells and clinical response (24). However, the highly overlapping ranges of the populations limit their use to identify patients with higher probabilities of responding to checkpoint inhibitors. Our data show how CD4^+^ and CD8^+^ CM and effector T cells are a bellwether of responses to checkpoint inhibitors, presumably because all of them contribute to the anti-tumor responses (25, 26). The integration of all these correlates of T cell status into a simple and novel parameter (CM/Eff T cell rations), allows a better separation between responders and non-responders and identification of those NSCLC patients most likely to experience clinical benefit from checkpoint inhibitor therapy.

There is a clear need to elucidate the mechanisms underlying primary resistance and short-lived clinical responses to checkpoint inhibitors. Our data suggest that the state of the T cell arm of the immune system, measured by the relative frequency of CM/Eff T cell ratios can be a contributing mechanism. Even more, improving the number of patients who can benefit from immune therapy requires a comprehensive analysis of the relative contributions of T cell subpopulations to anti-tumor responses. This challenge includes understanding whether a reduced naïve T cell repertoire contributes to functional T cell depletion, and the capacity of CM T cells to replenish the T cell repertoire (26). At a functional level, high levels of the pro-apoptotic molecule Bim in PD1^+^CD11a^+^CD8^+^ T cells of melanoma patients associate with shorter survival after anti-PD1 treatment, presumably because Bim may induce apoptosis of anti-tumor-specific T cells (27). Early pharmacodynamics effects of anti-PD1 associated with clinical benefit are the extent of expression of the proliferation marker ki67 in PD1^+^ T cells (28, 29) or of particular memory subtypes (30). An integrated analysis of these T cell subpopulations and their relationship to each other would provide a better understanding of the mechanisms behind primary resistance to anti-PD1 therapy. Along this line, a comprehensive analysis of the TCR repertoire together with gene expression in patients during checkpoint therapy would shed light on this particular question. Although there are still unanswered questions, this method to evaluate the immune system provides an easily accessible circulating biomarker to add to a comprehensive evaluation that already includes TMB and PDL1. Altogether, these assays may enable a better prediction of which patients will respond to checkpoint inhibitors, as well as those who may obtain more benefit from other agents.

## Ethics Statement

This study was carried out in accordance with the recommendations of The Moffitt Cancer Center’s Institutional Review Board and the Bristol-Myers Squibb Institutional Biosafety Committee. The protocol was approved by the Moffitt Cancer Center’s Institutional Review Board and the Bristol-Myers Squibb Institutional Biosafety Committee. All subjects gave written informed consent in accordance with the Declaration of Helsinki.

## Author Contributions

NM-O, LM, SK, PF, BK, CJ, JC, PK, PK, SB, SS, and SN planned and designed experiments; SK, PF, BK, CJ, VP, and MY performed experiments; NM-O, LM, SK, PF, BK, SS, MC, DG, RG, SB, and SN analyzed data; DG, PK, SL, and HD performed gene expression analysis; SS and SN provided intellectual input and helped preparing this manuscript; all authors approved the final version.

## Conflict of Interest Statement

Work funded by BMS. NM-O, LM, SK, PF, BK, CJ, MC, DG, VP, MY, RG, JC, PK, SS, SB, and SN are full-time BMS employees and own BMS stock.
