# Supplementary material for: Circulating T Cell Subpopulations Correlate With Immune Responses at the Tumor Site and Clinical Response to PD1 Inhibition in Non-Small Cell Lung Cancer
Source: Front Immunol. 2018 Aug 3;9:1613. doi: 10.3389/fimmu.2018.01613 (PMC6085412; doi:10.3389/fimmu.2018.01613)
Supplement: Supplementary file 1 [file Presentation_1.PDF]

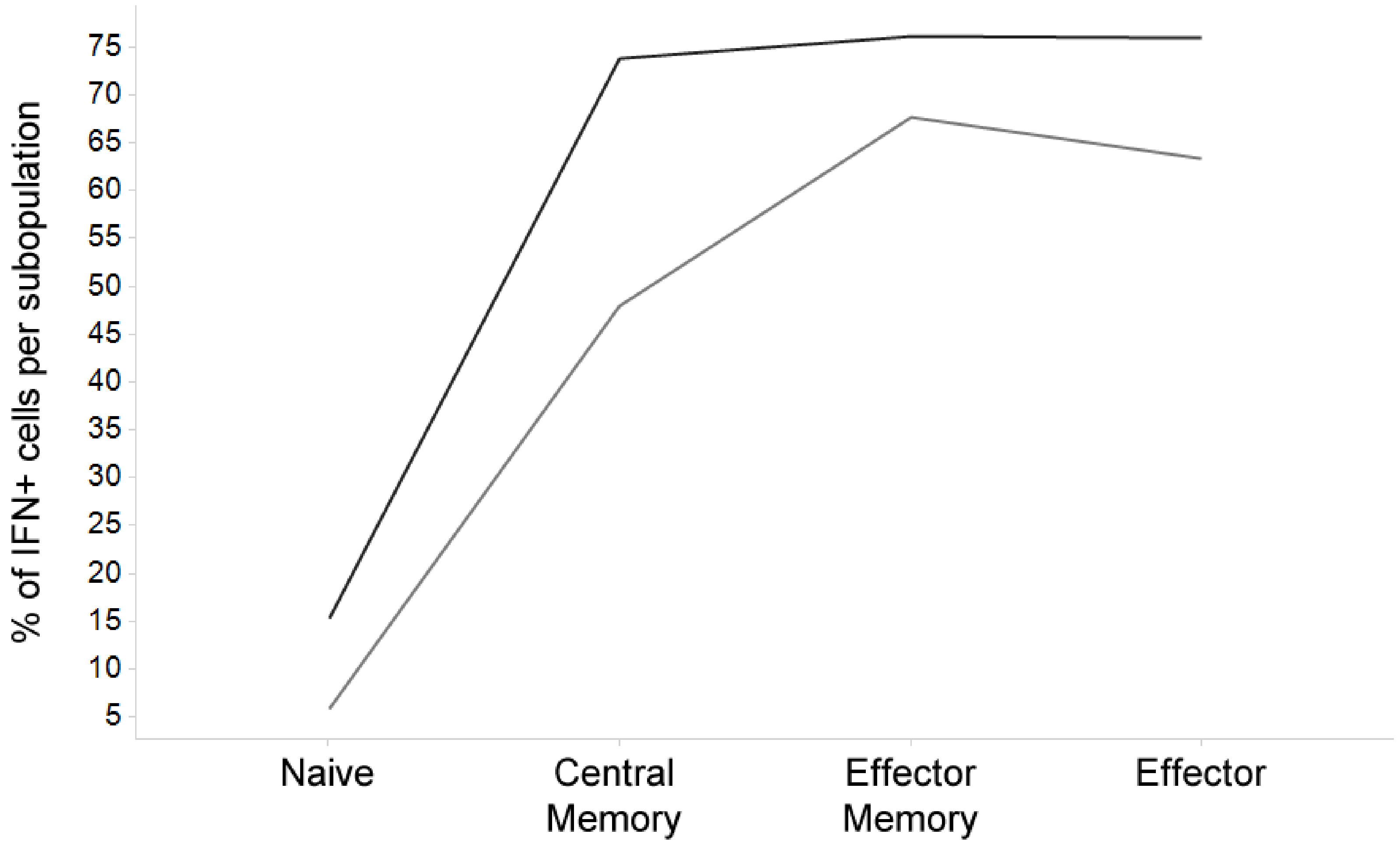

Supplementary Figure 1.
Interferon production by CD8 T cell subpopulations along differentiation in two control samples. PBMC were stimulated with phorbol-12-myristate 13-acetate (81 nM), ionomycin (1.33 μM) in the presence of Brefeldin A (5 μg/ml) (Cell Activation Cocktail; BioLegend) for 5 hours at 37°C. Samples were then stained for CD3 (Clone SK7, BV395), CD4 (SK3, BV496), CD8 (RPA-T8, APC-R700), CD45RO (UCHL1, BV786), and CCR7 (G043H7). Samples were treated with an intracellular fixation and permeabilization kit (BioLegend) following the manufacturer’s instructions to stain for IFN-γ (4S.B3, AX488) or an isotype control (MOPC-21, AX488).

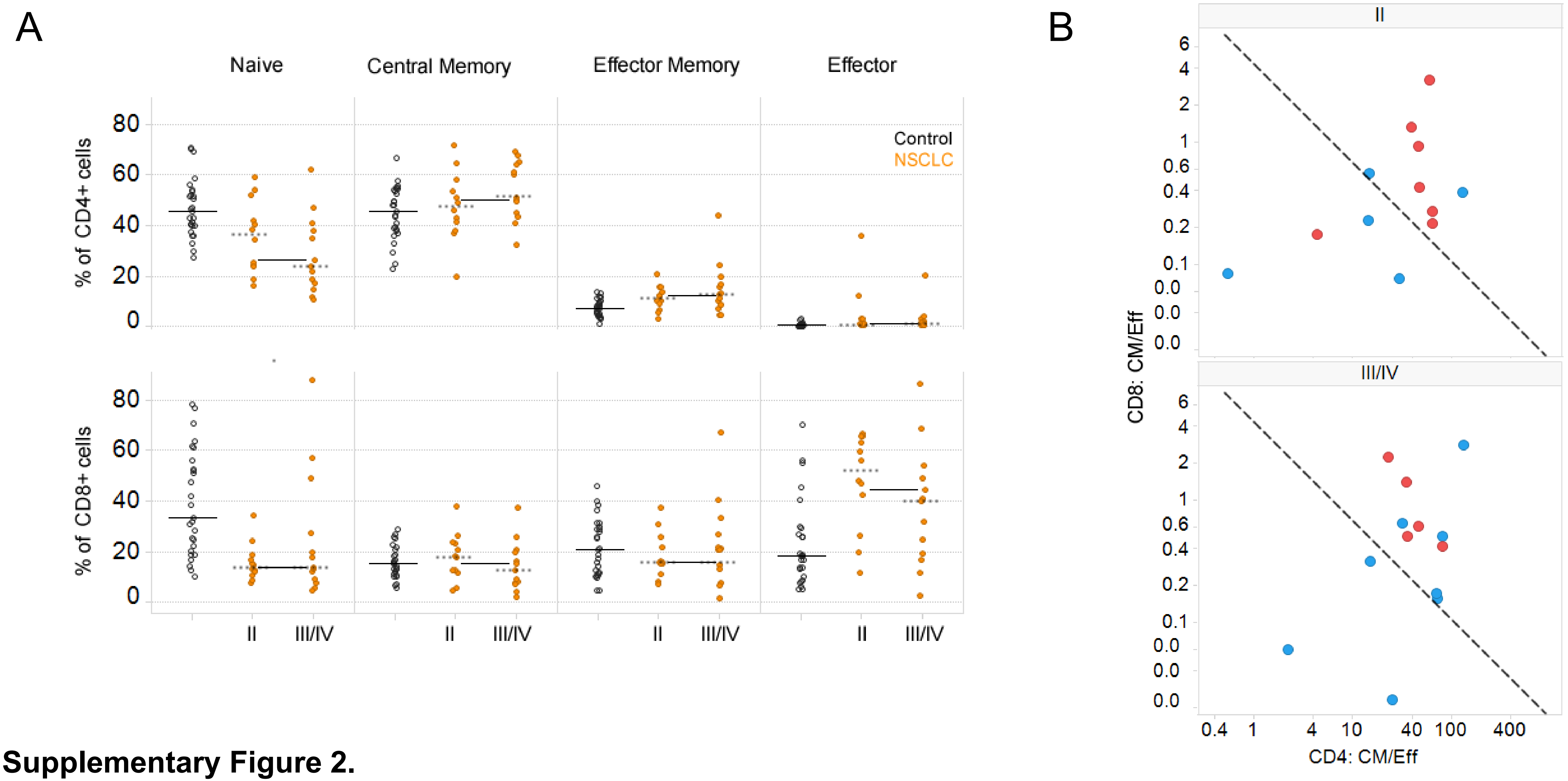

Supplementary Figure 2.
(A) Percentage of CD4 and CD8 T cell subpopulations in NSCLC separated by tumor stage (II vs III & IV). The continuous horizontal lines mark the median per each subpopulations, the dotted lines mark the median per subgroup.
(B) CM/Eff T cell ratios evaluated by tumor stage, (II vs III & IV), as described in the text. Chi-squared ns
